# ROS1-Rearranged Lung Cancer With Extensive Calcification on Computed Tomography: A Case Report

**DOI:** 10.7759/cureus.69083

**Published:** 2024-09-10

**Authors:** Junya Yoshioka, Kentaro Hizume, Shigeaki Iwatsubo, Kanoko Matsumura, Yasuhiro Funada

**Affiliations:** 1 Respiratory Medicine, Takatsuki General Hospital, Takatsuki, JPN

**Keywords:** adenocarcinoma, calcification, crizotinib, enutrectinib, lung cancer, ros1

## Abstract

Although calcified lung cancers are occasionally observed, they are quite rare. We report a case of extensively calcified lung adenocarcinoma with ROS1 fusions. A 57-year-old Japanese woman was initially diagnosed with adenocarcinoma based on aspiration cytology of an enlarged left supraclavicular fossa lymph node at the Department of Otorhinolaryngology at our institution. Subsequent chest imaging suggested primary lung cancer, leading to a referral to the Department of Respiratory Medicine. Computed tomography of the chest revealed a nodular shadow in the right S5 lobe and enlarged mediastinal lymph nodes with extensive calcification. A bronchoscopy with transbronchial lung biopsy of the right S5 nodule confirmed the diagnosis of adenocarcinoma. The biopsy specimen was analyzed using the AmoyDx® Pan Lung Cancer PCR Panel, which detected ROS1 fusions.

## Introduction

Calcification is typically associated with benign lesions; however, it can also appear in lung cancer, although less commonly [1]. Approximately 10% of lung cancer cases show calcification on computed tomography (CT) [2].

C-ROS oncogene 1 (ROS1) fusions are oncogenic drivers found in 0.7-1.7% of non-small cell lung cancers (NSCLCs) [3]. ROS1-rearranged lung cancer is more frequently observed in women, non-smokers, and younger individuals compared to other types of lung adenocarcinoma.

Few reports exist on lung cancer with extensive calcified lesions [4,5], and no cases have documented ROS1 fusions with such extensive calcification. Here, we present a case of extensively calcified lung adenocarcinoma with ROS1 fusions.

## Case presentation

**Figure 1:**
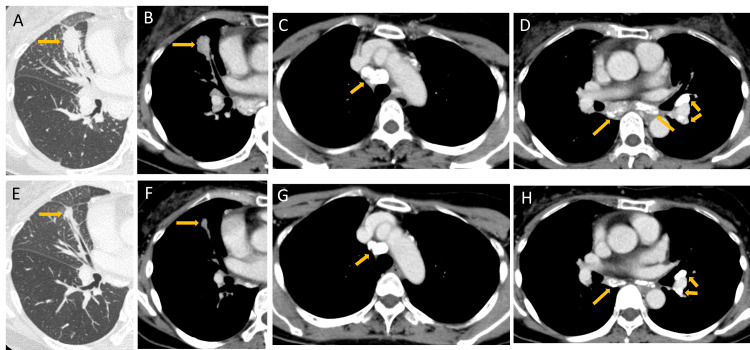
CT (A, B) CT showing a 25 × 14 mm nodular shadow in the right S5 lobe. (C, D) Hilar and mediastinal lymph nodes exhibiting extensive calcification. (E-H) Changes observed after one month of ROS1/MET inhibitor therapy. CT, computed tomography

A 57-year-old Japanese woman with no history of smoking was followed up by the Department of Otorhinolaryngology at our institution for a left parotid tumor. An enlarged left supraclavicular fossa lymph node was detected, and adenocarcinoma was diagnosed based on aspiration cytology. The tumor marker carcinoembryonic antigen level was very high at 2,058 µg/L. Subsequent chest imaging suggested that the lungs might be the primary site of the disease, leading to a referral to the Department of Respiratory Medicine. A CT scan of the chest revealed a 25 × 14 mm nodular shadow in the right S5 lobe, along with enlarged pulmonary hilar and mediastinal lymph nodes with extensive calcification (Figure 1).

**Figure 2:**
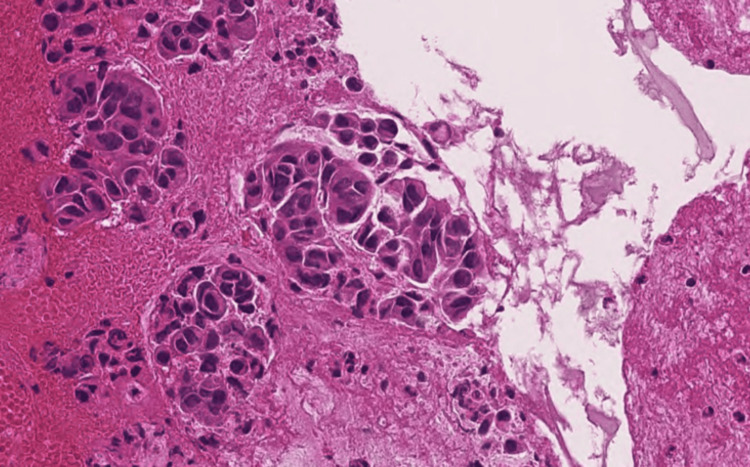
Pathological histology Adenocarcinoma with glandular cavity formation (H&E stain, ×20 magnification)

A transbronchial lung biopsy of the right S5 nodule was performed using bronchoscopy. Histopathological evaluation revealed an infiltrative proliferation of atypical cells with mutual adhesion and glandular cavity formation (Figure 2).

Immunohistochemical staining revealed positive thyroid transcription factor-1 and negative p40, consistent with lung adenocarcinoma. The specimen was analyzed using the AmoyDx® Pan Lung Cancer PCR Panel, which detected ROS1 fusions. Positron emission tomography-CT showed fluorodeoxyglucose accumulation in the contralateral hilar lymph node. The patient was diagnosed with stage IIIB lung adenocarcinoma (cT1cN3M0) according to the 8th edition of the American Joint Committee on Cancer staging system.

The patient was treated with the ROS1/MET inhibitor crizotinib (250 mg, twice daily) orally. No major non-hematological toxicities were associated with crizotinib. On day 12, the patient developed grade 2 neutropenia (Common Terminology Criteria for Adverse Events, version 5.0), prompting a dose reduction to 200 mg twice daily. Despite this, the patient developed grade 3 neutropenia, and crizotinib treatment was discontinued on day 26. CT imaging on day 27 revealed shrinkage of the primary lesion in the middle lobe and reduction in the mediastinal and hilar lymph nodes, confirming the efficacy of crizotinib treatment. However, calcified lesions persisted in the mediastinal and hilar lymph nodes. On day 36, treatment with the ROS1/tropomyosin receptor kinase inhibitor entrectinib (600 mg daily) was initiated.

## Discussion

Calcification within tumors has been observed on CT in approximately 10% of lung cancer cases [2]. Pathologically, 7.2% of lung adenocarcinoma cases exhibit psammoma bodies and other calcified structures [6]. Psammoma bodies are concentric lamellated calcified structures typically seen in papillary thyroid carcinoma, meningioma, and papillary serous cystadenocarcinoma of the ovary, but they are rarely reported in other neoplasms and non-neoplastic lesions [7]. In the present case, CT revealed extensive calcified lesions in the hilar and mediastinal lymph nodes, which were initially thought to be metastatic. However, no calcified lesions were found upon pathological examination, as the biopsy was performed on a lesion that did not show calcification on CT.

We report a case of lung adenocarcinoma with ROS1 fusion. Several studies have explored the association between calcified structures and genetic mutations in adenocarcinoma patients. Psammoma bodies and calcified structures were observed in 43% of ROS1-rearranged NSCLCs, compared to 11% of ALK-rearranged NSCLCs, and even fewer cases of EGFR-mutated (4%) and triple-negative tumors (5%) [8]. Thus, calcified lesions may be characteristic of lung adenocarcinomas with ROS1 fusion. Adenocarcinomas with psammoma bodies are often associated with tyrosine kinase inhibitor-targetable driver mutations in EGFR (69.8%), ALK (13.2%), and ROS1 (1.9%) [6], although the presence of psammoma bodies in ROS1-fused lung adenocarcinomas remains debated.

The mechanism behind tumor calcification is not fully understood. It can occur through necrotic degeneration (dystrophic calcification), the formation of psammoma bodies (concentrically laminated calcifications), or the engulfment of benign calcified lesions by carcinoma [5,6,9-11]. Mahoney et al. reported that calcification caused by tumor secretions is more common in adenocarcinomas [10]. Tumor secretions, such as mucin, contribute to calcification and the formation of psammoma bodies [4]. Given that ROS1-rearranged lung cancers often feature solid growth with signet ring cells or cribriform morphology with abundant extracellular mucus [12], these tumors may also present with calcifications similar to those observed in EGFR-mutated and ALK-rearranged lung cancers.

## Conclusions

Although cases of ROS1-rearranged lung adenocarcinomas with pathological psammoma bodies and calcified structures have been reported, there are no documented cases of lung adenocarcinomas with extensive calcification on CT. Here, we present a case of extensively calcified lung adenocarcinoma with ROS1 fusion. Additionally, lung adenocarcinomas with calcification may be associated with ROS1 fusions as well as EGFR mutations and ALK fusions.
